# Micro- and Nano-Structuring of Hydroxyapatite–MMT-Loaded Hydrogels for Bone Regeneration Applications

**DOI:** 10.3390/jfb17030121

**Published:** 2026-03-02

**Authors:** Inbar Eshkol-Yogev, Tom Hanoon Kogan, Inbar Levi, Maya Salman, Ofir Gariani, Meital Zilberman

**Affiliations:** 1 School of Biomedical Engineering, Tel-Aviv University, Tel-Aviv 69978, Israel; tomhanoon@mail.tau.ac.il (T.H.K.); inbarlevi1@mail.tau.ac.il (I.L.); mayushka26@gmail.com (M.S.); ofirgariany101@gmail.com (O.G.); 2 Unit of Periodontology, UCL Eastman Dental Institute, University College London, London WC1E 6DE, UK; i.yogev@ucl.ac.uk; 3 Department of Materials Science and Engineering, Tel-Aviv University, Tel-Aviv 69978, Israel

**Keywords:** nano-composite, micro-composite, MMT, gelatin, hydrogel, mechanical properties, physical properties

## Abstract

Bone regeneration focuses on the creation of functional tissue to repair bone defects. Creating a biodegradable scaffold hydrogel that combines a hemostatic agent with bioactive ceramics can afford the biological and mechanical benefits of both components. In the present study, we developed an injectable gelatin–alginate dual-composite hydrogel, loaded with two functional fillers: hydroxyapatite (HA) and the hemostatic agent montmorillonite (MMT). HA (microparticles and nanoparticles) was incorporated at concentrations of 10–30 mg/mL, with and without MMT at 20 mg/mL. The effects of functional fillers and their concentration on the microstructure and resulting physical and mechanical properties were studied, and a qualitative model summarising these effects was developed. All formulations exhibited clinically appropriate gelation times (5–29 s). n-HA significantly prolonged gelation time, reaching 29 ± 3 s at 30 mg/mL, while MMT reduced gelation time at all concentrations. The tensile strength of the unloaded hydrogel reached 20 kPa and increased to 57 kPa with 30 mg/mL of n-HA. The tensile strength even increased further with the addition of MMT (77 kPa). The results indicate that the combination of HA and MMT produced dual micro-composite hydrogels with moderate reinforcement, whereas the combination of n-HA and MMT generated dual nano–micro composites with combined reinforcing effects. The latter exhibited the highest strength and sealing ability while maintaining clinically relevant gelation times and controlled swelling behaviour. In conclusion, the combination of MMT with n-HA or HA enables the creation of functional hydrogels with controlled properties, tailored to specific applications in bone regeneration.

## 1. Introduction

Bone tissue regeneration remains a major challenge in orthopaedic and dental research due to the complex nature of native bone, which combines mineralised and organic components, vascularisation and cellular activity under continuous mechanical loading. Current treatments include various bone grafts (autografts, xenografts, allografts) and synthetic materials such as bioceramics and metals. Although they are in clinical use, none of these materials is ideal [1,2,3]. Autografts, although considered as the “gold standard”, are associated with a second donor site, patient morbidity and a limited amount of bone [2,3]. Allografts and xenografts carry the potential risk of disease transmission. Synthetic bone grafts (such as bioactive ceramics) provide an osteoconductive scaffold and can be produced in unlimited amounts; however, they lack osteoinductive properties [2,3].

The development of new biomaterials for bone repair must not only provide mechanical support but also present biological properties that promote cell adhesion, proliferation, and osteogenic differentiation. Hydrogels, particularly those derived from natural sources (such as gelatin), have emerged as promising biomaterials for bone regeneration due to their tunable properties, biocompatibility, and ability to mimic the extracellular matrix [4]. However, their relatively limited mechanical strength and lack of bioactivity often limit their use in load-bearing applications. For these reasons, natural hydrogels are frequently combined with other materials to create composite hydrogel systems [5].

Over the past few years, different biomaterials have been studied for bone tissue engineering, including bioceramics such as hydroxyapatite, beta-tricalcium phosphate, and bioglasses, to meet critical clinical needs in orthopaedic and dental fields [6].

Hydroxyapatite (HA), the primary mineral component of bone, is known for its excellent biocompatibility and osteoconductivity. With the development of nanoscale materials, nano-hydroxyapatite (n-HA) has been widely investigated as a scaffold for bone tissue engineering [7,8]. n-HA can promote mineralisation and is a common choice as a bone graft substitute and bone filler [9]. Nanocomposites based on layered silicates, particularly montmorillonite (MMT), have been studied when incorporated in various synthetic and natural polymers for several applications. They demonstrated significant improvements in the physical and mechanical properties compared to the polymers alone. MMT introduces layered microstructures that enhance polymer–filler interactions, improve cohesive and sealing properties, and reduce swelling [10]. In addition to strengthening the mechanical properties, the use of nanoclay composites, particularly MMT, represents a promising approach for wound-healing and bone-regeneration applications [11,12,13,14]. The combination of hydroxyapatite and MMT within a hydrogel matrix may provide a synergistic approach to enhance the biological and mechanical performance of bone scaffolds [15]. Despite extensive independent investigation of HA and MMT in bone-related applications [7,8,11,16,17], studies combining these two functional fillers within a single injectable hydrogel system remain limited [13,18].

In the current study, we investigated unique injectable hydrogels loaded with functional fillers for bone regeneration applications. The target application of the biomaterial is as an injectable hydrogel for bone grafting and bone tissue engineering, intended to fill irregular bone defects and support early-stage healing. Gelatin and alginate, the main components of our hydrogels, are well-known, highly biocompatible natural polymers. The hydrogel was crosslinked using the carbodiimide N-(3-dimethylaminopropyl)-N-ethylcarbodiimide hydrochloride (EDC). The functional fillers hydroxyapatite and MMT were incorporated into the hydrogel matrix to create dual-composite scaffolds. Based on our previous studies of MMT-containing gelatin–alginate hydrogels, a fixed MMT concentration was selected in the present work to specifically evaluate the added value of HA incorporation rather than to re-optimise MMT content [10,19].

The rationale for the current study is to combine the advantages of enhanced mineralisation in ceramics with the biocompatibility and biodegradability of the hydrogel, and to assess the contributions of both functional fillers (HA and MMT) to the hydrogel’s properties. This concept of an injectable, natural-based, dual-composite hydrogel for bone regeneration is novel and has been scarcely explored. This work focuses on establishing the structure–property relationship of the hydrogels and the effects of the fillers’ concentrations and HA particle size (micro-HA vs. nano-HA) on the physical and mechanical properties, with a concluding formulation-properties effects model.

## 2. Materials and Methods

### 2.1. Materials

Coldwater fish skin “type A” gelatin (G7041), alginic acid sodium salt (A1112), the crosslinking agent N-(3-dimethy laminopropyl)-N-ethylcarbodiimide hydrochloride (EDC), hydroxyapatite (reagent grade powder synthetic), and nanohydroxyapatite (nanopowder synthetic, <200 nm particle size) were purchased from Sigma-Aldrich, Rehovot, Israel. Sodium montmorillonite (Cloisite Na^+^) was purchased from BYK (Gonzales, TX, USA).

### 2.2. Preparation of the Composite Hydrogel

Preparation of the hydrogel was based on the preparation of two aqueous solutions, a polymer solution and a crosslinker solution, mixed just before use. The polymer solution was prepared by dissolving gelatin, alginate, and HA or n-HA in double-distilled water and heating it to 60 °C. In the second stage, MMT was added to part of these formulations. Gelation in this system is achieved via chemical crosslinking rather than thermal gelation, enabling injectability and in situ hydrogel formation. The crosslinker solution was obtained by dissolving EDC in double-distilled water. Based on our previous studies, the unloaded formulation (control) used in the current study contained 400 mg/mL gelatin, 10 mg/mL alginate, and 20 mg/mL EDC. HA/n-HA concentrations were 10, 20 and 30 mg/mL. MMT concentration was 20 mg/mL. The MMT concentration was selected based on our previous studies, in which this MMT content demonstrated the most favourable balance between gelation time, viscosity, and biocompatibility when evaluated independently.

**Figure 1:**
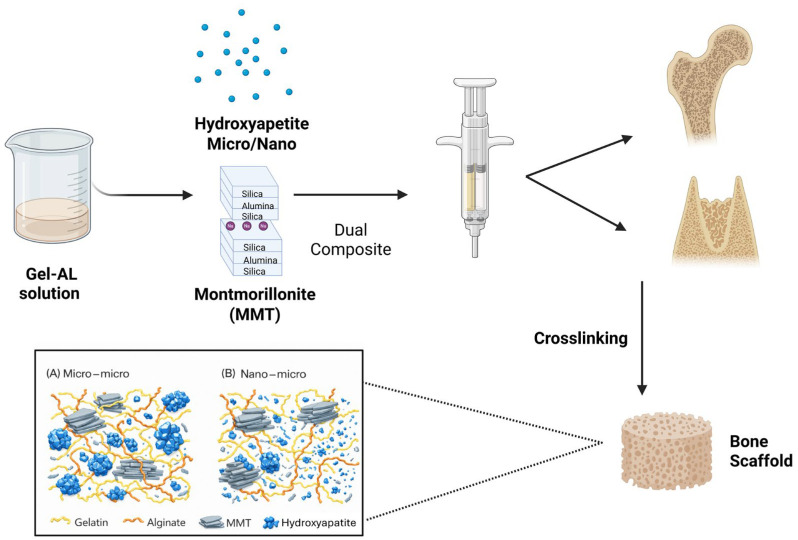
The preparation process from the Gel-Al solution, the dual syringe and potential use as a bone scaffold. This figure was created using BioRender.com, accessed on 31 January 2026.

In all experiments, the hydrogel was prepared and applied using a double syringe with a static mixer at a 4:1 volume ratio (Mixpac L-System, Sulzer, Switzerland), which ensured consistent mixing of the polymer and crosslinker solutions. The polymer solution, including both fillers, was loaded into the large chamber of the double syringe, and the crosslinker solution was loaded into the small chamber. The preparation process is described in Figure 1.

### 2.3. Evaluation of the Physical Properties

#### 2.3.1. Gelation Time

Using a double syringe with a static mixer (Mixpac L, Sulzer, Switzerland) at a 4:1 volume ratio, approximately 1 mL of the polymer solution and 0.25 mL of the crosslinker solution were poured onto a 1.6 cm-diameter plate. The solutions were mixed at 300 rpm using a 1.4 cm magnetic stir bar and incubated at room temperature.

Gelation time (i.e., crosslinking time) was defined as the time required for the magnetic stir bar to stop rotating after mixing the polymer and crosslinker solutions, indicating gel formation.

#### 2.3.2. Swelling Ratio and Weight Loss

To determine the hydrogels’ swelling ratio and weight loss, the hydrogels were poured into 6.2 × 6.2 × 3.5 mm^3^ silicon moulds. After crosslinking (about 5 min), the samples were removed and transferred to 24-well plates.

The initial weight of each sample was recorded (*Wi*). The samples were then immersed in 1.5 mL of water and incubated at 37 °C and 100% relative humidity for 2 and 24 h. After incubation, excess water was removed, and the hydrogels were gently blotted with Kimwipes and weighed again (*Ws*). Subsequently, the samples were dried for 24 h and 48 h and weighed again to obtain the final weight (*Wf*). The swelling degree and weight loss of the formulations were calculated using the following equations:

Swelling degree: (*Ws* − *Wi*)/*Wi* × 100% (1)

Weight loss: (*Wi* − *Wf*)/*Wi* × 100% (2)

#### 2.3.3. Microstructural Characterisation

The microstructure of the hydrogel loaded with both fillers was investigated to characterise the dispersion of the additives within the hydrogel matrix. For this purpose, cubic specimens of approximately 0.5 mL were dried with silica gel at 37 °C for 48 h, freeze-fractured at −80 °C, and their cross-sections were observed using an Environmental Scanning Electron Microscope (Quanta 200 FEG ESEM, FEI Company, Hillsboro, OR, USA) in a high-vacuum mode, with an accelerating voltage of 10 kV. In addition, elemental composition was characterised using EDS.

### 2.4. Evaluation of the Composite’s Mechanical Properties

#### 2.4.1. The Bulk Properties of the Hydrogel

**Figure 2:**
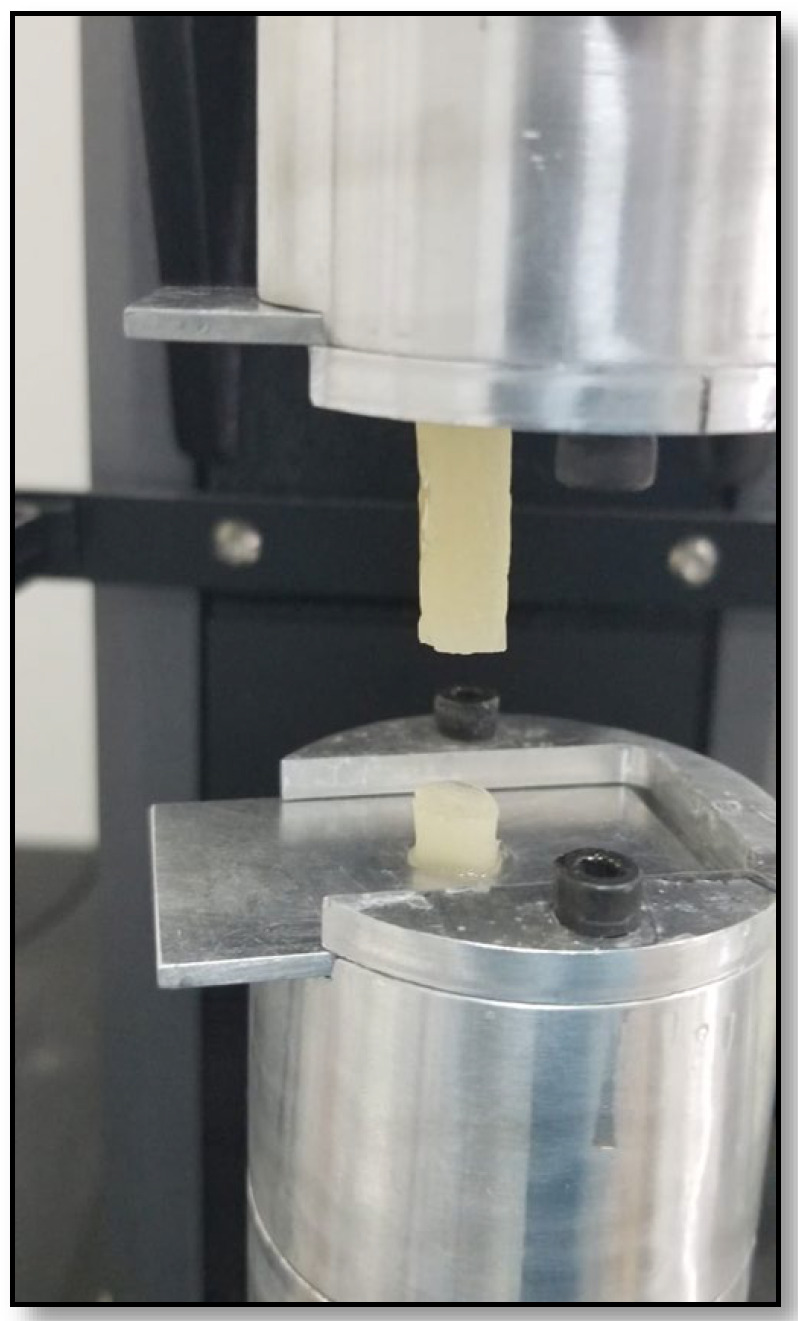
Cylindrical samples in the Instron Universal Testing Machine (Model 5500, Instron Engineering Corp., Norwood, MA, USA).

Cylindrical specimens (8 mm diameter, 40 mm height) were fabricated using a silicone mould and tested 24 h after casting. They were subjected to tensile displacement at a rate of 5 mm/min until failure using a Instron Universal Testing Machine (Model 5500, Instron Engineering Corp., Norwood, MA, USA) to determine Young’s modulus and tensile strength. For each formulation, 5 to 7 samples were evaluated (Figure 2). 

#### 2.4.2. Burst Strength Measurements

The burst pressure test evaluates the capacity of a hydrogel to adhere to tissue surfaces and provide effective sealing, primarily reflecting its cohesive strength. Burst strength was determined using a custom-built mechanical burst apparatus in accordance with the standard test method for Burst Strength of Surgical Sealants (ASTM F2392-04) [20]. This method measures the maximum pressure the hydrogel can withstand at the site of tissue leakage. A collagen casing (S1 Fibran, Sant Joan de les Abadesses, Spain) containing a standardised 3.0 mm diameter defect served as the tissue substrate. Approximately 0.5 mL of hydrogel was applied to seal the defect, forming a layer with an approximate thickness of 1 mm. The prepared sample was mounted in the testing device, and pressure was gradually applied until failure occurred. The pressure at hydrogel rupture was recorded as the maximum burst pressure. At least 10 replicates were tested for each formulation.

### 2.5. Cytotoxicity Evaluation

In order to evaluate the cytotoxicity, human dermal fibroblast cell cultures were exposed to hydrogel extracts for specific periods of time, as described in the ISO 10993 Standard (parts 5 & 12) for biological evaluation of medical devices [21].

#### 2.5.1. Preparation of Hydrogel Extract

Hydrogel samples were made in a 7.0 × 7.0 × 3.5 mm^3^ silicon mould. After crosslinking, they were removed and dried overnight. The sterilisation process was done by gamma irradiation. Hydrogel extracts were obtained by immersing the sterilised samples in a culture medium at a concentration of 60 mg/mL and incubating them for 24 h at 37 °C.

#### 2.5.2. Cell Cultures

Human dermal fibroblasts were isolated from neonatal foreskin tissue (HFFn). Cells were thawed and expanded in 75 mm^3^ culture flasks containing Dulbecco’s Modified Eagle Medium supplemented with 10 percent fetal bovine serum and 1 percent penicillin–streptomycin–nystatin. Cultures were maintained at 37 °C in a humidified atmosphere with 5 percent CO_2_. Upon reaching approximately 70 percent confluence, cells were detached using trypsin A solution and seeded into 96-well plates at a density of 5000 cells per well. After 24 h of incubation, the culture medium was removed and replaced with 0.1 mL of hydrogel extract per well. Cells cultured without extract served as the negative control. The cells were then incubated for an additional 72 h. Eight replicates were performed for each formulation.

#### 2.5.3. Alamar Blue Assay for Cell Viability

Cell proliferation and viability in the presence of hydrogel extracts were assessed using the Alamar Blue assay. The assay was performed 24 and 48 h after exposure to the hydrogel extracts. The procedure included replacing the original medium with 0.25 mL of fresh medium containing 10% (*v*/*v*) Alamar Blue and incubating the cells for 4 h. Subsequently, 100 μL duplicates from each well were transferred into a 96-well plate for spectrophotometer analysis (Spectra Max 340 PC384, Molecular Devices, San Jose, CA, USA). The percentage of Alamar Blue reduction was calculated according to the manufacturer’s protocol. Cell viability following exposure to hydrogel extracts was determined by comparison with control cells not exposed to the extracts, allowing evaluation of hydrogel cytotoxicity.

### 2.6. Statistical Analysis

All data were processed using GraphPad Prism version 10.6.1, for Windows, GraphPad Software, www.graphpad.com. Statistical comparisons between more than two groups were performed using ANOVA (with Tukey–Kramer post hoc). A value of *p* < 0.05 was considered statistically significant. Error bars indicate the standard deviation. In all figures, statistically significant differences are indicated with an asterisk.

## 3. Results and Discussion

The effects of the formulation parameters on the microstructure and on the most relevant resulting mechanical and physical properties were elucidated in this study and are presented here in a “chronological sequence”, i.e., the gelation time results are presented first because it is the time required to convert a polymeric solution into a hydrogel. We then present the microstructural characteristics of the dual-composite hydrogels created and, finally, the properties most relevant for bone regeneration applications. Examining the composite hydrogel in tensile mode demonstrates its sensitivity to contact between the hydrogel and the fillers; therefore, the tensile properties were measured. In addition, it was characterised for burst strength, which demonstrates its sealing ability, and for swelling and weight loss kinetics, which strongly affect its functioning.

### 3.1. Gelation Time

**Figure 3:**
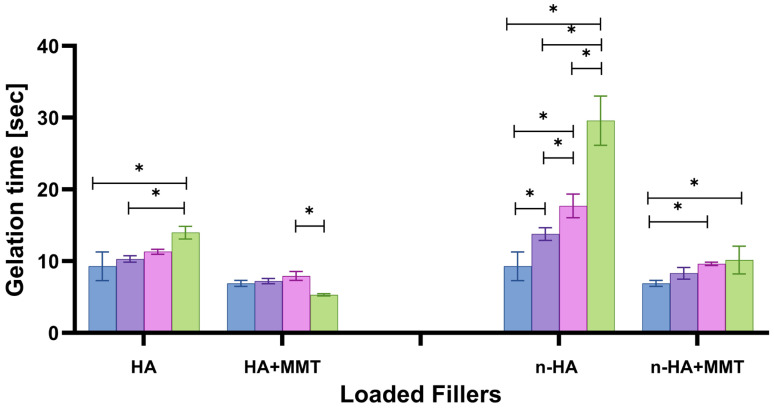
Gelation time of the hydrogel as affected by the HA and n-HA concentrations. (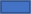 0 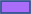 10 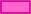 20 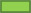 30 mg/mL) with and without the addition of MMT (20 mg/mL). 0 mg/mL for both HA and n-HA corresponds to the unloaded hydrogel, which serves as the control. Significant differences between the groups are marked with an asterisk (*p* < 0.05).

In injectable hydrogels for bone regeneration, it must be possible to control the gelation rate to enable appropriate surgical handling. If the gelation time is too fast, the surgeon will not be able use it, and if it is too slow, functionality will be achieved after a relatively long time. The optimal gelation time for most applications is 5–60 s [22]. The gelation time of all formulations tested in the present study is within this range (Figure 3). The gelation time of HA-loaded hydrogels was only slightly influenced by the HA concentration. However, the gelation time of the n-HA-loaded hydrogels was dramatically affected, increasing by 200% at the maximal n-HA concentration of 30 mg/mL (29 ± 3 s). The differences between HA- and n-HA-loaded hydrogels were statistically significant between the groups at all concentrations (*p* < 0.05). These results indicate that the nano-sized HA particles interfere with the curing process of the gelatin–alginate formulation. The slower gelation rate of n-HA-loaded hydrogels is attributed to the nanoparticles’ higher surface area. A larger amount of the HA’s Ca^+2^ ions are exposed to the hydrogel when HA is in nano-size and can create bonds with the R-COO^–^ ions in gelatin or alginate, based on opposite charges. This may “compete” with the EDC crosslinker, thereby decreasing the gelation time.

The hemostatic agent MMT was loaded into the hydrogel at a relatively high concentration (20 mg/mL). The effect of MMT on gelation time is shown in Figure 3. In contrast to the effect of HA, MMT incorporation slightly decreased the gelation time of HA-loaded hydrogels. When loading the n-HA formulations with MMT, the gelation time decreased significantly for relatively high n-HA concentrations (20 and 30 mg/mL). The MMT layered structure enables dispersion within the hydrogel and accelerates its physical crosslinking effect. In fact, our results show that n-HA and MMT have opposite effects on the hydrogel’s gelation time, with n-HA exerting a stronger impact. It should be noted that the gelation time of the n-HA-loaded formulations (13–29 s) is more suitable for bone regeneration applications than that of the unloaded formulation (9 s), enabling the surgeon more time to handle and shape the hydrogel properly. It can be further tuned to meet clinical needs, a significant benefit in bone regeneration procedures.

### 3.2. Microstructural Characterisation (ESEM/EDS)

**Figure 4:**
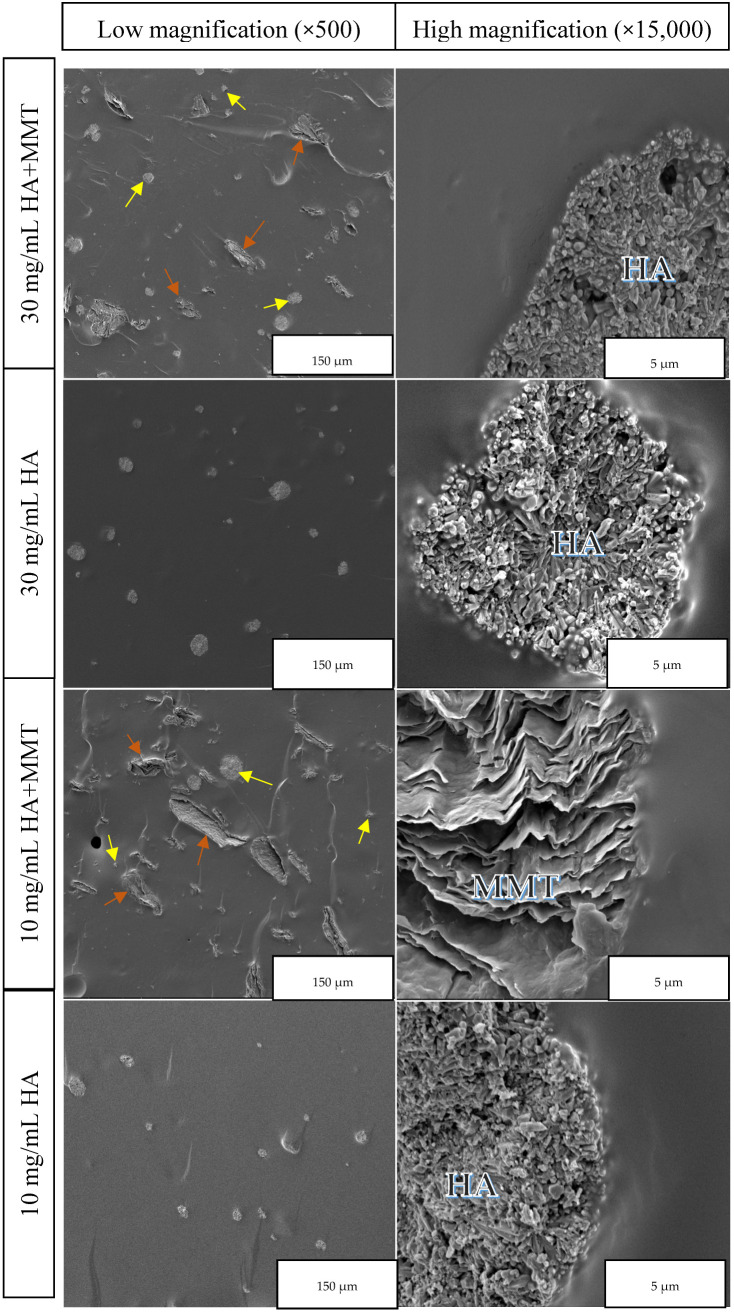
ESEM micrographs of hydrogels containing HA (yellow arrow), with and without MMT (orange arrow), at low and high magnifications.

The ESEM observations reveal the dispersion of MMT and hydroxyapatite within the hydrogel matrix, as well as their interfacial interaction with the polymer network. It also enables the observation of differences in microstructure resulting from the various filler concentrations and sizes. MMT, a layered clay material, can be clearly identified in the 10 mg/mL HA + MMT sample, as shown in Figure 4 (×15,000). It appears to be relatively uniformly distributed as flat, layered microstructures with widths ranging from a few micrometres to several tens of micrometres. A slight penetration of the polymeric matrix into the MMT layers was also observed, contributing to strong mechanical interlocking. A small amount of MMT is likely mixed at the nanoscale, as supported by the EDS results shown below. The absence of distinct nanoscale MMT features suggests that the material did not fully integrate into a nanostructured form, likely due to a mild mixing of the formulation.

The interface between the MMT and the hydrogel appears well integrated, indicating excellent wettability. HA is also well dispersed within the hydrogel. At lower magnifications, it is arranged in relatively spherical microstructures with an average size of approximately 10 µm, as seen in Figure 4 across all concentrations, including in the presence of MMT. At higher magnifications, its characteristic crystalline structure is especially evident in Figure 4 for the 30 mg/mL HA sample (×15,000). The interface in this case also appears well integrated, indicating excellent contact with the hydrogel.

**Figure 5:**
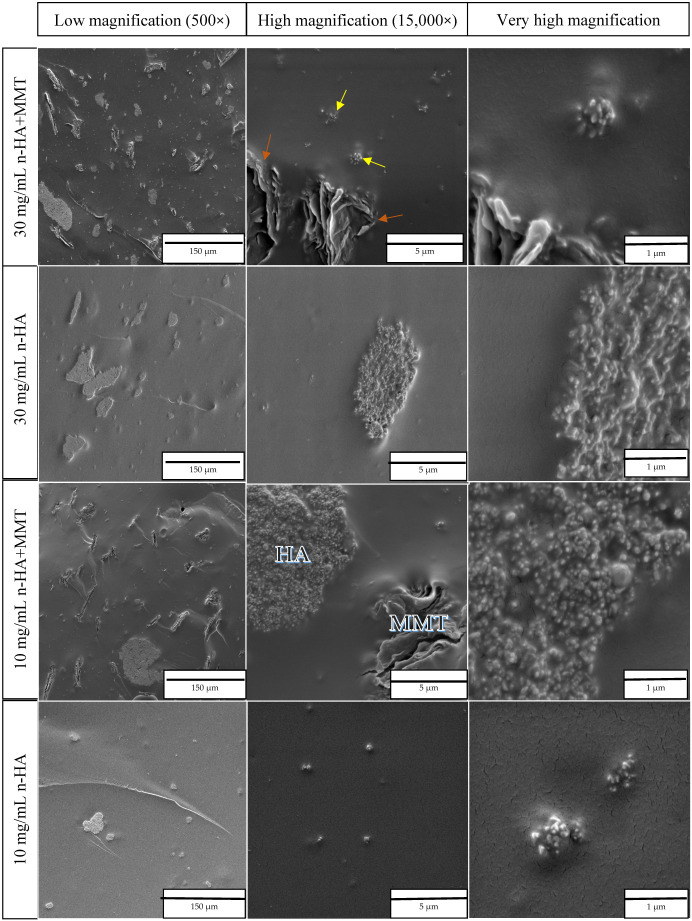
ESEM micrographs of hydrogels containing n-HA (yellow arrow), with and without MMT (orange arrow), at low, high and very high magnifications.

In the case of n-HA, a homogeneous distribution throughout the hydrogel is likewise observed; however, the n-HA is arranged in a broad range of structures, from the nanometre scale up to several tens of micrometres. Notably, at the higher n-HA concentration of 30 mg/mL, a greater number of the larger-scale structures are observed (Figure 5), suggesting concentration-dependent aggregation behaviour. Here as well, the interface with the hydrogel indicates excellent wettability. When examining the formulations containing MMT in combination with either HA or n-HA, no direct interaction between the two functional fillers could be observed. Each of them appears to be dispersed independently within the hydrogel matrix.

**Figure 6:**
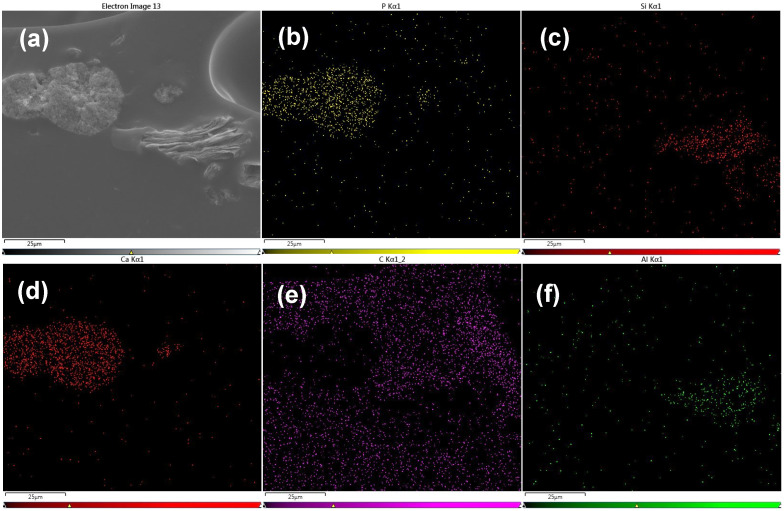
EDS micrographs of hydrogels containing HA and MMT. (**a**) SE Image, (**b**) EDS map of Phosphorus, (**c**) Silicon, (**d**) Calcium, (**e**) Carbon, and (**f**) Aluminium.

**Figure 7:**
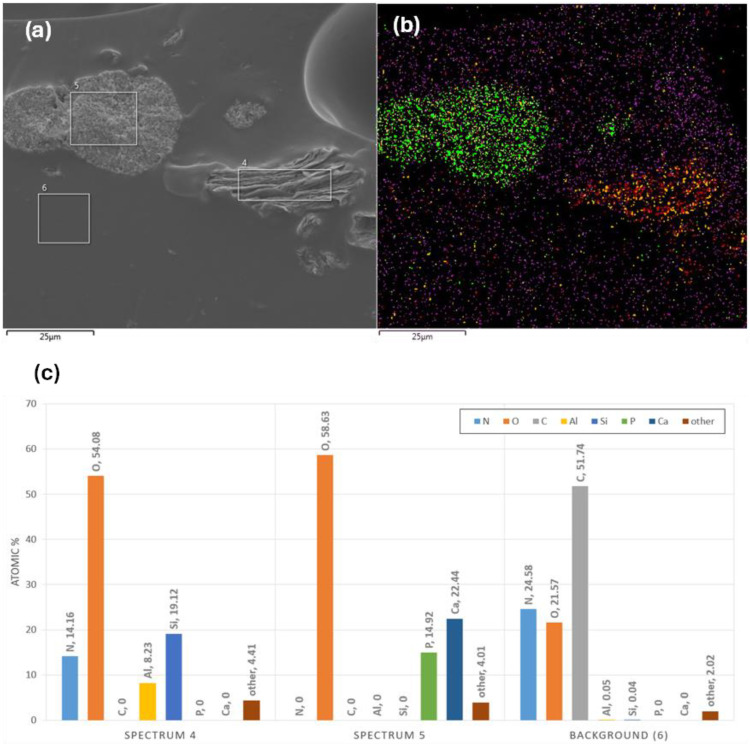
EDS results of hydrogels containing HA and MMT. (**a**) SE Image showing the analysed regions, (**b**) EDS map of Phosphorus (yellow), Silicon (red), Calcium (green), Carbon (purple), Aluminium (orange), and (**c**) corresponding EDS spectra presented as bar charts for the three marked regions.

EDS analysis of the micrographs enables the determination of the relative signal intensities of different elements, which leads to verification of the presence of HA and MMT in the sample. In Figure 6a, the particle on the left exhibits a crystalline morphology; inspection of the corresponding EDS spectra (Figure 6b,d) shows that the two main components are Calcium and Phosphorus, confirming its identification as HA. In contrast, the particle on the right in Figure 4 displays a layered structure and, based on the EDS spectra (Figure 6c,f), shows that the two main components are silicon and aluminium, consistent with its classification as MMT. Examination of the homogeneous background indicates a composition dominated by carbon, which fits the gelatin-based hydrogel matrix. In Figure 7, the EDS elemental signals are overlaid (with colour modifications) on the corresponding SEM image to enable improved visual correlation and comparison. Low silicon and aluminium signals detected in the hydrogel matrix (Figure 7c) can indicate some nanostructuring of the MMT.

### 3.3. Tensile Mechanical Properties

**Figure 8:**
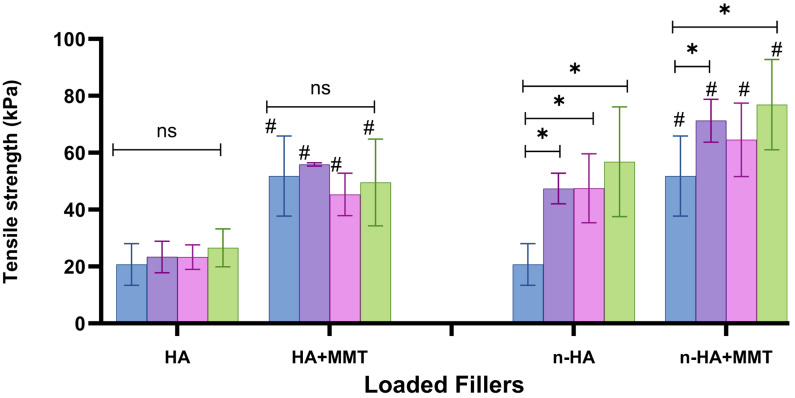
The tensile strength of the hydrogels as affected by the HA and n-HA concentrations. (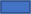 0 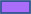 10 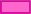 20 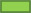 30 mg/mL), with and without the addition of MMT (20 mg/mL). 0 mg/mL for both HA and n-HA corresponds to the unloaded hydrogel, which serves as the control. Significant differences between the non-loaded formulation are marked with (*). Significant differences (*p* < 0.05) between the groups (with or without MMT) are marked with (#), NS, not significant (*p* ≥ 0.05).

The effects of HA and n-HA, with and without the addition of MMT, on the tensile strength are presented in Figure 8. The addition of HA had almost no effect on tensile strength compared with the unloaded hydrogel control (0 mg/mL, blue bar). In contrast, the incorporation of n-HA particles enabled achieving a tensile strength approximately 3 times higher than that of the unloaded basic formulation, and for example, 30 mg/mL n-HA increased tensile strength from 20 kPa (unloaded) to 57 kPa (30 mg/mL). A further increase in strength was obtained by incorporating MMT particles, reaching a maximum of 75 kPa for the formulation containing 30 mg/mL n-HA and 20 mg/mL MMT. Incorporation of MMT into the HA-loaded formulation also resulted in an almost 2-fold significant increase in the tensile strength.

Incorporation of strengthening particles into a polymer or hydrogel usually increases its strength, both in micro- and nanostructuring, with a greater effect in nanostructuring [23]. In the current study, a substantial strengthening effect was observed for the n-HA-loaded hydrogel. In contrast, the HA-loaded hydrogel did not exhibit any strengthening effect, although the HA was spread into micro-particles in the hydrogel.

In addition to the hemostatic effect of MMT incorporation, its microstructural effect results in higher tensile strength for both HA and n-HA formulations (Figure 8). The hydrogel–MMT particles’ wettability probably contributes to this behaviour.

**Figure 9:**
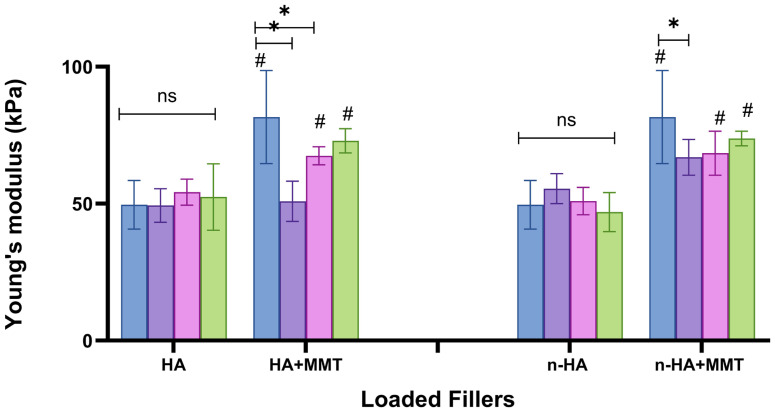
The Young’s Modulus of the hydrogels as affected by the HA and n-HA concentrations (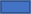 0 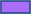 10 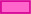 20 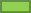 30 mg/mL), with and without addition of MMT (20 mg/mL). 0 mg/mL for both HA and n-HA corresponds to the unloaded hydrogel, which serves as the control. Significant differences between the non-loaded formulation are marked with (*). Significant differences (*p* < 0.05) between the groups (with and without MMT) are marked with (#), NS, not significant (*p* ≥ 0.05).

The Young’s modulus as affected by the addition of HA and n-HA, with and without the addition of MMT, is presented in Figure 9. Contrary to the tensile strength, Young’s modulus was barely affected by increasing the concentration of HA or n-HA. n-HA incorporation increased the tensile strength, and we therefore also expected an increase in Young’s modulus, which indicates resistance to elastic deformation. Since this did not occur, the increased strength appears to have happened in parallel with an increase in the maximal strain, suggesting that we did not lose ductility when incorporating n-HA particles. It should also be noted that relatively low HA and n-HA concentrations were used in the current study. The addition of MMT to the HA-loaded formulations resulted in a slight increase in Young’s modulus, from approximately 50 kPa across all studied formulations to 65–80 kPa in most of them (Figure 9). This corresponds to the increase in tensile strength observed with MMT incorporation (Figure 8). In fact, incorporation of both bioactive agents, the bone mineral HA and the hemostatic agent MMT, results in dual composite structures.

The HA/MMT-loaded hydrogel can be described as a dual micro-composite, in which both the HA and the MMT particles primarily generate microstructural features within the polymer matrix. In this system, the mechanical properties are mainly governed by the micro-scale reinforcements. In contrast, the n-HA/MMT-loaded hydrogel is a nano–micro dual composite, in which n-HA introduces nanoscale structural effects while MMT primarily contributes to microscale features. The combined action of these micro- and nanoscale reinforcements enhances the cohesive strength of the bulk hydrogel, resulting in a significant improvement in its mechanical strength and stiffness. These findings are entirely consistent with the excellent interfacial quality observed in the SEM images, as high-quality hydrogel fillers’ wettability, along with effective interfacial interactions, directly support the observed increase in mechanical strength.

Several studies combined polymers with n-HA to create composite hydrogels, combining the desirable properties of both polymers and n-HA, including enhanced mechanical properties. Li et al. developed polyacrylamide/n-HA composite hydrogels that exhibited higher tensile and compressive strengths and greater ductility than the corresponding hydrogels [24]. Ribeiro et al. presented a novel composite hydrogel consisting of silk fibroin and n-HA. In this composite, increasing the n-HA concentration improved its mechanical properties [7]. This improvement in mechanical properties was also shown for other systems based on natural polymers, such as gelatin [25], agarose [26], and chitin [27]. Our study is unique in that it elucidates the combined effects of n-HA or HA with the effects of micro-structures obtained due to the incorporation of the hemostatic agent MMT.

### 3.4. Burst Strength (Sealing Ability)

**Figure 10:**
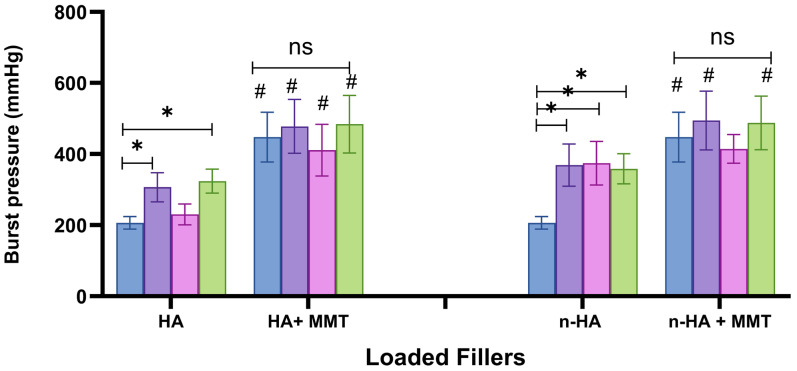
The burst strength of the hydrogels as affected by the HA and n-HA concentrations (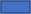 0 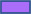 10 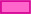 20 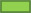 30 mg/mL), with and without addition of MMT (20 mg/mL). 0 mg/mL for both HA and n-HA corresponds to the unloaded hydrogel, which serves as the control. Significant differences between HA and n-HA are marked with (#). Significant differences between the non-loaded hydrogel and HA/n-HA are marked with (*), NS, not significant (*p* ≥ 0.05).

The sealing ability of a biomaterial is essential for bone regeneration. It can prevent epithelial proliferation into the wound area, stabilise the coagulum, and support tissue regeneration [28]. The effect of HA and n-HA, also combined with MMT, on the burst strength (i.e., on the maximal pressure that the hydrogel can withstand), is presented in Figure 10. The burst strength of the unloaded formulation was 206 mmHg. Addition of HA or n-HA increased the burst strength to 323 and 358 ± 42 mmHg, respectively. These results support the assumption that nanoparticles are more effective than microparticles in enhancing the cohesive strength of the hydrogel and, consequently, improving sealing and adhesion to the tissue, thereby enhancing bone regeneration.

The addition of MMT further increased the burst strength of both types of systems. HA-loaded hydrogels reached a maximal burst strength of 480 mmHg, and n-HA-loaded hydrogels reached a maximal burst strength of 498 mmHg (Figure 10). This effect of MMT incorporation is attributed to a strong interaction between the gelatin and MMT, especially hydrogen interactions between carboxylate groups in the gelatin molecules and hydroxyl groups in the MMT. Moreover, MMT is an expanding layered silicate that can significantly increase the silicate’s surface area and thus the number of interactions.

### 3.5. Swelling Degree and Weight Loss

The ability of a scaffold to retain water is an essential indicator of its efficacy in tissue engineering. The degree of swelling and the weight loss of hydrogels are critical physical parameters that indicate the network density between the polymer chains formed by the crosslinking reaction. A low swelling ratio indicates a dense, crosslinked polymer network, which reduces water molecules’ ability to enter the hydrophilic regions of the polymer. As a result, less water can penetrate the hydrogel structure. In scaffolds, increased swelling enables efficient nutrient transport and increases the likelihood of cell infiltration in a three-dimensional pattern, mimicking cell growth under physiological conditions in vivo [29]. However, if the swelling degree is too high, the material may lose its ability to function with appropriate mechanical properties.

**Figure 11:**
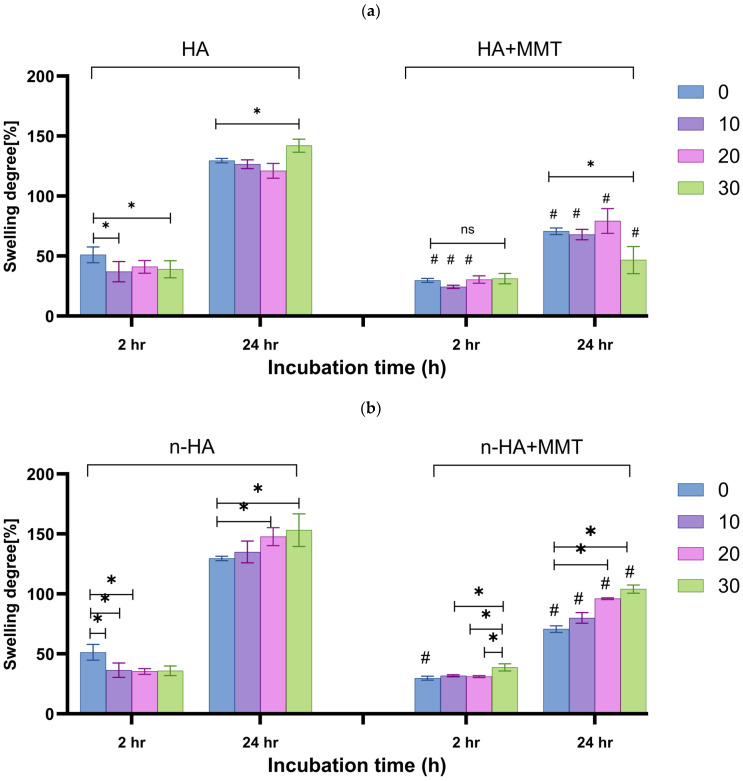
The swelling degree of the hydrogels as affected by the incubation time, for various hydroxyapatite concentrations (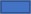 0 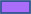 10 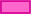 20 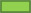 30 mg/mL): (**a**) HA, and (**b**) n-HA loaded hydrogel with and without addition of MMT (20 mg/mL). 0 mg/mL for both HA and n-HA corresponds to the unloaded hydrogel, which serves as the control. Significant inter-group differences are marked with (*). Significant intra-group differences are marked with (#), NS, not significant (*p* ≥ 0.05).

The swelling degree of the composite hydrogel as affected by the HA type, concentration, and MMT incorporation is presented in Figure 11. After two hours of incubation, both HA and n-HA loaded formulations presented a slightly lower swelling degree than the unloaded formulation (~50% swelling). Differences between the HA and the n-HA hydrogels were non-significant (*p* > 0.05). After 24 h of incubation, the HA-loaded formulations exhibited swelling degrees in the range of 120–140%, and the n-HA-loaded formulations exhibited swelling degrees in the range of 135–150%. The higher degree of swelling associated with hydrogels containing n-HA may be due to the hydrophilicity caused by the free—OH groups of HA and higher surface area of the nanoparticles, which accelerate diffusion of water into the particles. A similar trend was reported previously by Dhivya et al. [30].

The addition of MMT decreased the hydrogel’s swelling in all formulations (Figure 11), with a more pronounced effect after 24 h. After 24 h of incubation, the HA + MMT-loaded formulations exhibited a swelling degree range of 50–75%, and the n-HA + MMT-loaded formulations exhibited a swelling degree range of 70–100%. While the increase in n-HA led to greater swelling due to its hydrophilicity, caused by the free –OH groups, the incorporation of MMT had an inverse effect by absorbing part of the water that penetrated the polymeric matrix, resulting in less swelling. Our results show that MMT has a greater impact on swelling than n-HA.

**Figure 12:**
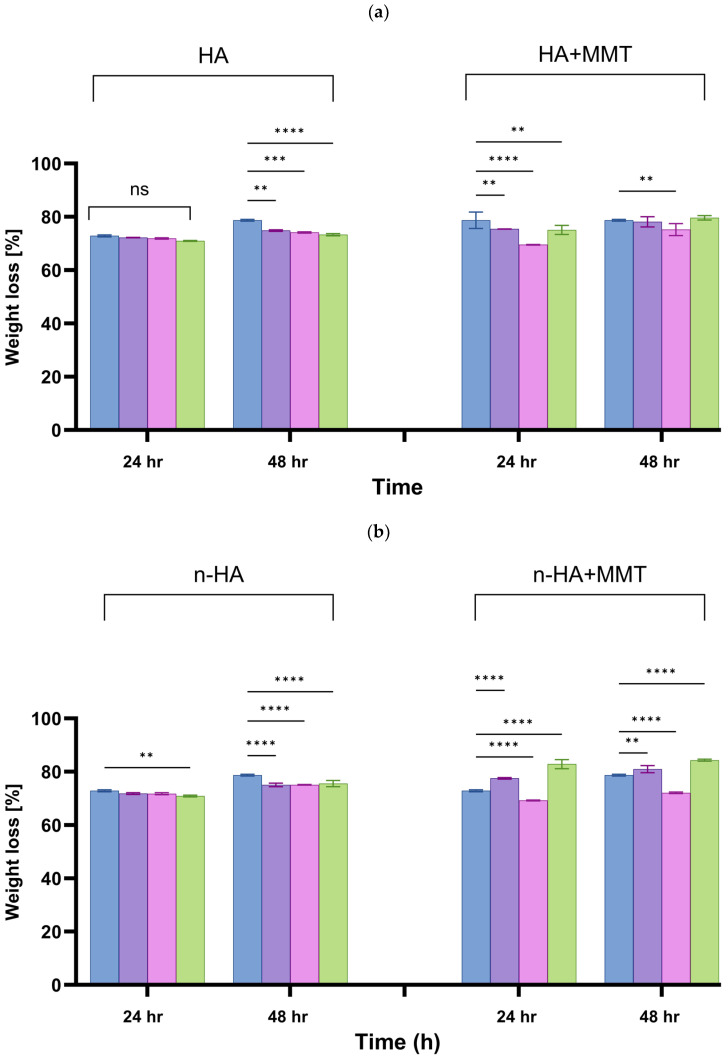
The weight loss degree of the hydrogels as affected by the incubation time, for various hydroxyapatite concentrations (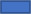 0 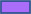 10 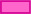 20 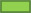 30 mg/mL): (**a**) HA, and (**b**) n-HA loaded hydrogel with and without addition of MMT (20 mg/mL). 0 mg/mL for both HA and n-HA corresponds to the unloaded hydrogel, which serves as the control. Significant inter-group differences are marked with (*), NS, not significant (*p* ≥ 0.05).

The degree of weight loss in hydrogels indicates the ease with which polymer chains detach from the network. The weight loss of the composite hydrogel, as affected by the HA type and concentration, is presented in Figure 12. All studied formulations exhibited approximately 70% weight loss after 24 h of incubation, with a slight additional increase after 48 h. For HA-based hydrogels, weight loss at 24 h showed no significant differences among concentrations, whereas clear, concentration-dependent increases were observed at 48 h. In contrast, n-HA-based hydrogels displayed significant differences already at 24 h, which became more pronounced after 48 h. The incorporation of MMT increased overall weight loss for both HA and n-HA systems at both time points and enhanced the differences between concentrations. These results indicate that weight loss is primarily a result of time-dependent release of non-crosslinked polymer chains, while HA/n-HA type and MMT incorporation modulate the extent and kinetics of this process.

### 3.6. Cell Viability and Cytotoxicity

**Figure 13:**
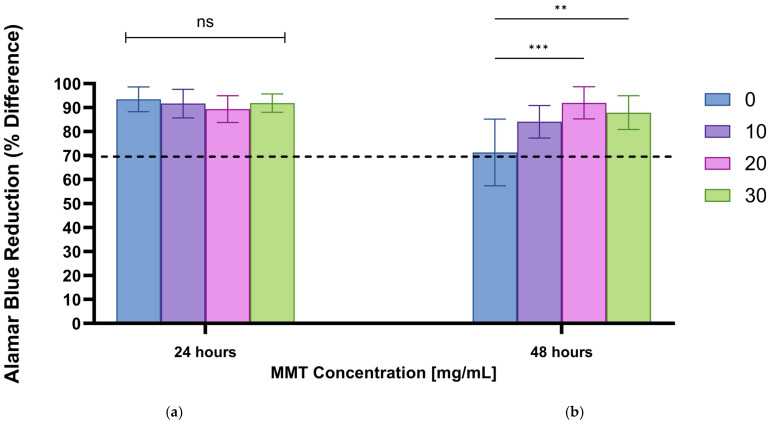
The effect of the exposure of unloaded hydrogel with and without different MMT concentrations (mg/mL) on the Alamar blue reduction, indicating fibroblast cell viability, after (**a**) 24 h and (**b**) 48 h. Significant differences are marked with an asterisk, NS, not significant (*p* ≥ 0.05).

The Alamar Blue assay was performed on human fibroblasts involved in the wound-healing process to evaluate cell viability in the presence of the hydrogels. As HA-based formulations were evaluated in our previous studies [17], the present work focused on MMT-containing hydrogels, which represent the most cytotoxic component and are therefore more critical to assess. Formulations that result in a decrease in viability of more than 30% are considered cytotoxic. The results (Figure 13) indicate that after 24 h, cells in the presence of all hydrogel formulations exhibited high viability (above 90%; Figure 13). After 48 h, cell viability decreased in some formulations (Figure 13) but remained above 70%. While no significant differences were observed at 24 h, after 48 h, the 20 mg/mL MMT-containing formulation showed the least Alamar Blue reduction, supporting its selection as the optimal concentration for this study and indicating an optimal balance between cell compatibility and functional MMT incorporation.

## 4. Summary and Limitations

**Figure 14:**
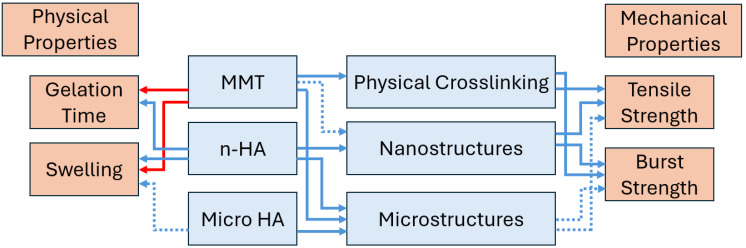
A schematic representation of a qualitative model describing the effects of the hydrogel’s components on the mechanical and physical properties. The blue/red arrows represent a case in which an increase/decrease in a specific parameter results in an increase/decrease in the following parameter. Dashed lines represent a more moderate response.

In the current study, we developed and characterised novel dual-nanocomposite hydrogels for bone regeneration. They were loaded with two functional fillers, hydroxyapatite (microparticles and nanoparticles) and the hemostatic agent montmorillonite. The effects of these fillers and their concentration on the physical and mechanical properties were studied, and a qualitative model summarising these effects is presented in Figure 14.

HA exhibited only a mild influence on the hydrogel’s behaviour across all tested properties. The gelation time remained similar to that of the basic formulation, indicating minimal interference with the EDC crosslinking reaction. ESEM images showed HA dispersed as spherical microstructures, and the hydrogel demonstrates good contact with them, with no gap appearing. However, this did not translate into a mechanical strengthening effect. The tensile strength and Young’s modulus were essentially unchanged, and burst pressure increased only moderately. Swelling and weight loss were slightly reduced at early time points but remained comparable to those of the base hydrogel. Overall, HA acted primarily as a biologically active filler rather than a structural reinforcement agent.

In contrast, the n-HA had a much more pronounced effect on all hydrogel properties. Its high surface area significantly slowed the gelation process, probably due to competing ionic interactions with the gelatin and alginate chains. Structurally, n-HA formed a broad distribution of features ranging from true nanoparticles to larger aggregates. The nanoscale reinforcement strongly enhanced the mechanical performance: the tensile strength increased up to threefold, the burst pressure rose substantially, and the good hydrogel particles’ wettability observed by SEM supported this behaviour. Incorporation of n-HA also increased swelling after 24 h, due to its high hydrophilicity, and promoted greater water uptake than the HA systems. Despite these effects, the weight loss remained generally similar to that of the basic formulation, with slight reductions at early stages. Thus, n-HA acted as a true reinforcing nanofiller, improving the cohesive properties of the hydrogel while maintaining overall network integrity.

MMT produced microscale layered structures within the hydrogel, despite being a nanomaterial, and showed excellent interfacial bonding with the hydrogel matrix. MMT slightly accelerated gelation and significantly decreased swelling by absorbing part of the penetrating water and tightening the network. When combined with HA, MMT transformed the system into a dual micro–micro composite, resulting in noticeable improvements in tensile strength, Young’s modulus, burst pressure, and reduction in swelling relative to HA alone.

When combined with n-HA, MMT produced a dual nano–micro composite that exhibited the strongest performance among all formulations. The microstructures originating from MMT and the nanoscale reinforcement provided by n-HA acted synergistically to dramatically increase tensile strength and modulus, yielding the highest burst pressures. These synergistic effects align with SEM observations showing excellent filler–matrix interfaces and independent yet complementary dispersion of the two fillers, with no evidence of direct interaction between them.

The use of relatively low filler concentrations was aimed at strengthening the hydrogel without compromising the hydrogel’s resorbable nature. In the context of resorbable bone scaffolds, maintaining a high polymer and filler density may hinder tissue ingrowth and reduce the available space required for new bone formation. Therefore, relatively lower filler content was selected to preserve scaffold porosity and resorbability while minimising potential cytotoxic effects associated with higher particle loading. Despite the low particle density observed in the SEM images, measurable and reproducible effects on gelation time and mechanical properties were achieved, demonstrating that even limited amounts of HA and MMT can effectively modulate the hydrogel’s material behaviour.

While the study shows promising results, some limitations should be acknowledged. The study presents a preliminary investigation and focuses mainly on the material characterisation of the composite hydrogel. While previous studies from our group have assessed HA and MMT individually, both in vitro and in vivo [17,19,31], the combination of HA and MMT has not yet been evaluated biologically. Therefore, this work emphasises the physical and mechanical properties together with the microstructure, without in vitro or in vivo testing of the dual composite. Future studies will include comprehensive biological evaluations, including cell compatibility and bone regeneration models, to assess the combined effects of HA and MMT in bone tissue engineering applications.

## 5. Conclusions

In conclusion, the distinct behaviours of HA, n-HA, and MMT, especially their combined effects, enable the design of hydrogels with a broad spectrum of tunable mechanical and physical properties. By adjusting filler type, scale, and concentration, these dual-composite hydrogels can be tailored to specific clinical needs, offering a versatile and powerful platform for injectable bone regeneration scaffolds. Future in vivo preclinical studies using the composite hydrogel would be the next step toward its clinical use.

## Data Availability

The original contributions presented in the study are included in the article, further inquiries can be directed to the corresponding author.
